# Strengthening global health cooperation-insights from worldwide WHO collaborating centres

**DOI:** 10.1016/j.hpopen.2025.100158

**Published:** 2025-12-19

**Authors:** Sophia Achab, Benedetto Saraceno

**Affiliations:** a Reconnecte Specialized Treatment Program Head, Addiction Division, University Hospitals of Geneva, Switzerland; b Psychological and Sociological Research Unit Head, WHO Collaborating Centre for Training and Research in Mental Health, Department of Psychiatry, Faculty of Medicine, Geneva University, Switzerland; c Lisbon Institute of Global Mental Health, Lisbon, Portugal

**Keywords:** WHO, Collaborating centers, Strategy, Management, Leadership, Qualitative research

## Abstract

**Background:**

WHO Collaborating Centres (WHO CCs) are entities designated to provide scientific or technical support to the World Health Organization (WHO) in specific areas. They play a crucial role in global public health efforts; however, their leadership and sustainability present multiple complex challenges.

**Objectives:**

The aim of this analysis is to identify the strategic and management challenges faced by WHO CCs and to provide expert recommendations to enhance their sustainability. This study focuses on leadership capabilities as a key driver of successful global health cooperation.

**Methods:**

The study employed a three-phase approach: (1) a conceptual framework for strategic management of WHO CCs, (2) empirical testing through qualitative interviews with WHO CCs directors worldwide, and (3) development of actionable recommendations. A flexible sampling strategy was used, including convenience, purposeful, and database-based sampling to ensure diversity across regions, institution types, and health topics.

**Results:**

WHO CCs are manageable organizations but face unique strategic (mission alignment, core values, vision), and management challenges (project management, financial constraints, communication barriers).

Long-lasting WHO CCs were led by directors whose leadership practices aligned with established models of effective leadership (e.g., self-awareness, strategic positioning, clear, and engaging vision). Key threats included a lack of resources, recognition, or project management skills, misaligned strategic vision, and communication gaps with WHO or the host institution.

**Conclusion:**

WHO CCs are vital to global health but require structured strategic management and leadership development. Their strategic management must take into account both the similarities and differences with other organizations. Expert recommendations include securing financial resources, improving WHO- WHO CCs communication, and fostering leadership skills to ensure sustainability and impact.

## Highlights

• Successful collaboration requires values’ alignment, engaging vision and leadership.
• Careful choice of WHO CC Director with international credibility in the specific topic.
• Deep commitment, high emotional intelligence, effective leadership practices.
• WHO CC’s hosting institution should provide support and resources to the workplan.
• Joint-venture WHO- WHO CCs, symmetric partnership, country-level support.

## 1 Introduction

### 1.1 Background

WHO Collaborating Centres (WHO CCs) are specialized institutions designated by the World Health Organization (WHO) to provide scientific or technical support in specific health areas [1]. They are critical to global public health and contribute to program implementation, guideline development, disease monitoring, standardization, dissemination, and training [1].

However, their sustainability is currently threatened by complex leadership and management challenges, further compounded by their dual affiliation with WHO and their host institutions.

WHO CCs are functionally linked to WHO through a joint work plan agreed upon for each designation period. They are required to allocate resources to this plan and adhere to WHO's operating rules. Successful collaboration, essential for re-designation and sustainability, relies on the completion of the work plan [1]. An objective indicator of successful collaboration between WHO and its WHO CCs is the duration of their active status.

### 1.2 Problem statement

As of February 2020, the number of active WHO CCs has declined significantly, with 2,267 WHO CCs having lost their designation since WHO’s inception [2]. This trend raises concerns about the loss of expertise and the reduction of economic resources essential for global health collaboration [2]. Two hypotheses explain this decline: (a) suboptimal collaboration conditions, and (b) WHO’s strategic downsizing to address overlaps or to rebalance the geographical distribution [3].

This study focuses on the first hypothesis, exploring how strategic and management challenges—particularly leadership—impact WHO CCs’ sustainability.

### 1.3 Objectives

This study aims to identify strategic and management challenges faced by WHO CCs; to test hypotheses about core values, vision, and strategic positioning using a qualitative approach; and finally, to develop actionable recommendations from expert WHO CCs’ directors to enhance sustainability.

## 2 Methods

### 2.1 Study design

A qualitative, phenomenological approach was used to explore WHO CCs’ directors’ experiences. The study comprised three phases:

1. **Conceptual Framework:** Theoretical analysis of strategic management in WHO CCs.
2. **Empirical Testing:** Interviews with WHO CCs’ directors to validate the framework.
3. **Recommendations:** Actionable strategies co-constructed with WHO CCs directors .

### 2.2 Sampling strategy

A three-stage sampling process [4] ensured diversity:

1. **Convenience sampling** for geographically accessible directors.
2. **Purposeful sampling** to include high-yield informants from professional networks.
3. **Database-based sampling** to cover underrepresented regions/topics and target WHO CCs that have been active for a minimum of two years. Out of a total of 46 invited directors, 13 accepted to partcipate to the study (28 % response rate). Susequently, data from ninecountries and five WHO regions were collected (Table 1).

**Table 1:** Sample description (n = 13).

Gender	Number of years of experience directing a WHOCC	WHO Region	Number of years of designation of the WHOCC	Number of designation periods of the WHOCC
M	2	EURO	2	1
M	4	EURO	11	3
M	6	AMRO	6	2
M	7	EMRO	28	7
M	7	WPRO	7	2
M	8	WPRO	30	8
M	10	EURO	10	3
F	10	AMRO	28	7
M	11	EURO	11	3
F	13	SEARO	28	7
M	18	WPRO	14	4
M	19	EURO	19	5
M	25	WPRO	25	6

WHO was not directly involved in the survey, but the study utilized the publicly available WHO CCs database to identify and contact potential participants. The invitations were sent to WHO CC directors via email .

### 2.3 Data collection

Semi-structured interviews were conducted face-to-face (n = 5), over the phone (n = 1), and via email (n = 7). Interviews were audio-recorded, transcribed verbatim, and analyzed. An interview guide (Annex) with standardized questions on: Strategic challenges (core purpose, vision, threats), and management challenges (project management, financial constraints, communication) was used for all WHO CCs directors. It included a collection of theWHO CCs directors’ recommendations for WHO CC sustainability.

### 2.4 Data analysis

Thematic analysis followed these steps:

1. **Coding:** Transcripts were coded for “core purpose,” “leadership skills,” “threats,” etc.
2. **Theming:** Patterns were identified to reflect WHO CCs directors’ perspectives.
3. **Saturation:** No new themes emerged after 13 interviews, confirming data sufficiency [5].

### 2.5 Ethical considerations

Anonymity and confidentiality were ensured. Participants provided informed consent for audio recording.

## 3 Results

### 3.1 Conceptual framework: theoretical analysis of WHO CC strategic management

Strategy is defined as creating a unique and valuable position through a specific set of activities. There is an inextricable link between the development and execution of strategy and leadership [6]. The role of a leader within strategic plan development is to have a sustainability goal for the institution. This involves developing a plan that integrates specific features of the organization, such as missions, core purpose, vision, strategic options, opportunities, and threats [7].

The conceptual framework for strategic success, used in the present analysis, was developed by Collins and Porras and emphases managing continuity and change [7]. It is based on managing (a) **core ideology** elements that should never change such as **core purpose** and **core values**, while taking advantage of needed changes and progress to accomplish what an organization aspires to, and (b) an **envisioned future**. Therefore, a clear consideration of missions and functional scope is essential [7]. WHO CCs have concrete goals and expected deliverables formalized in a four-year work plan. However, operational effectiveness does not guarantee an organization's sustainability. To last, a WHO CC must identify and preserve its core purpose, which is the organization's most fundamental reason for existing. This core purpose inspires change and success by fuelling people's motivation to carry out the organization's activities. Core values are the essential and enduring tenets of an organization [7]. WHO has five core values: trust, commitment to excellence, integrity, collaboration, and caring [8]. WHO CCs are expected to align with these values and never compromise them under any condition. The hypothesis is that enduring WHO CCs align with WHO's core values and use them as guiding principles in their collaborative activities.

The envisioned future is a big and bold objective that takes 10 to 30 years for the organization to attain [7]. It should inspire, catalyse commitment, and stimulate progress within the organization [7]. Vivid descriptions of this Big, Hairy, Audacious Goal (BHAG) convey conviction and passion and translate into a clear and engaging mental image of the organization's future [7]. Designing a strategy for a WHO CC involves clarifying it’s position on strategic factors for key stakeholders (WHO and the host institution). Two strategic options that drive institutional sustainability are “**fit**” and “**strategic positioning**” [6]. The ”fit“ involves making activities interact and reinforce one another, while ”strategic positioning“ involves using distinctive activities to achieve sustainability in a competitive context [6]. Designation by WHO as a WHO CC represents a formal, time-limited opportunity for strategic positioning in a specific area of expertise, which is critical for the designated health organization to capitalize upon.

Building a strategic plan for an organization requires defining **key actions** needed to achieve strategic goals [9]. This involves moving from effective strategy conceptualization to practical actions to guarantee successful strategy implementation [9]. Successful strategic leadership involves having organizational-level **strategic thinking** from the early stages of strategy design [9]. Identifying threats that could face strategy execution, whether internal or external, is also crucial [9]. A WHO CC is a particular health organization at the crossroads of WHO and the institution of affiliation at a strategic and operational level. No executive requirements (leadership or management proficiency) are made by WHO for the profile of the head of the designated entity [3]. However, **leadership skills** are paramount for ensuring the long-term strategic existence of a WHO CC. Leadership is a key role in strategy design and execution but is often subject to misconceptions. Effective leadership is not about innate vision or charisma but about acquired skills to sharpen and stretch [10]. Leadership is about coping with rapid changes and adapting to them accordingly, while management is about coping with complexity, and bringing forth order and predictability [10]. Effective leaders exhibit eight main practices: asking “what needs to be done?” and “what is right for the enterprise?”, developing and revising action plans, taking responsibility for decisions and communication, focusing on opportunities rather than problems, running productive meetings, and thinking and saying “we” rather than “I” [11]. Other important characteristics of effective leaders include self-awareness, self-regulation, motivation, empathy, and social skills [12].

WHO CCs are also engaged in implementing the agreed work plan in a timely manner, which requires planning specific projects and coordinating resources to achieve them within WHO’s defined timeframe. **Project management** is a codified process involving planning, build-up, implementation, and closeout, with clear role definition, team member selection, and priority setting [13].

A WHO CC participates in various activities jointly agreed upon during the four-year designation period. **Funding** these activities is a daily challenge for a WHO CC. The major factors include the lack of financial support from WHO, the need to cover resources and costs of the work plan implementation through the affiliation institution’s core budget or extra-budgetary resources, and the limited funding sources due to WHO's Framework of Engagement with Non-State Actors (FENSA), which controls for potential conflicts of interest arising from financial links with the private sector [1].

**Communication** is a crucial leadership challenge in organizations, both internally (inspiring, motivating, integrating differences, resolving conflicts, coordinating, and aligning people with the organization's vision and decisions) and externally (persuading partners and stakeholders and gaining visibility) [14]. Effective leaders must manage communication carefully, precisely, and regularly to achieve success and performance. WHO CCs also share these communication issues, with specific factors regarding WHO’s communication rules, such as actively engaging in a collaborative network of WHO CCs, sharing expertise, opportunities, and building synergies through a regular, effective, and efficient communication system [1].

### 3.2 Empirical testing: interviews with WHO CC directors to validate the framework

Warranted anonymity of interviews was a cornerstone of the present data collection due to the sensitivity of the explored field and the need for confidentiality and discretion. Audio recording of face-to-face and phone call interviews was done solely upon agreement from participating directors. They were transcribed verbatim by the interviewer and complemented with handwritten notes. Written answers were considered as transcripts.

Data collected were coded according to our theoretical framework. Coding consisted of “core purpose”, “envisioned future”, “strategic positioning”, “actionable strategies”, “leadership skills”, “management challenges” and “recommendations for practice”. The interpretation of the narratives was conducted from a phenomenological standpoint to reflect each key director’s perspective. Themes were created as the transcripts were analysed, progressing from the first to the last interview. Directors were assigned a key informant identifier, indicated by a number used in superscript [1], [2], [3], [4], [5], [6], [7], [8], [9], [10], [11], [12], [13] in the results presented.

The inclusion period lasted from June 2019 to February 2020 and yielded a total of 13 interviews, of which five were face-to-face, one was a phone call, and seven were conducted via Interviews were conducted in three languages (French, English, and Spanish). Audio recordings lasted a total of 6 h.

Responding directors were mostly male (85 %), with a mean of 10 years (2–25) of experience directing a WHO CC (Table 1). They represented WHO CCs having 17 years of activity (2–30), and continuous labelling for one to eight periods (4–32 years). Five WHO regions were covered: EUR, WPR, AMR, SEAR, and EMR. The countries represented were Australia, Canada, Chile, China, Italy, Japan, Morocco, Switzerland, and Thailand. Collaborating topics covered neurology, mental health, e-health, nutrition, food safety, primary care, addiction medicine, humanitarianism, psychosocial health, and infection control.

Data saturation was achieved: all continents were represented, all country income groups were included, a broad range of leadership experience within WHO CCs was represented, gender diversity was ensured, diverse collaboration fields were explored, and various host institutions were included (hospitals, universities, and research centres). The sample size for this qualitative research was sufficient due to data satisfaction [14], meaning that the saturation point was reached after a few interviews and no additional information was obtained from subsequent interviews regarding the opinions and practices explored. According to Marshall et al.'s [15] requirements for qualitative sampling, sufficient adequate data had been collected for a detailed analysis, and findings could be then transferable to other WHO CCs.

Throughout the interviews, there was a strong sentiment of commitment, idealism, and passion from the WHO CCs’ directors. This was despite the reality of the challenges and complexity of their tasks. Open discussions with them revealed various examples of innovation, solution-oriented leadership, and self-motivation.

**Fig. 1:**
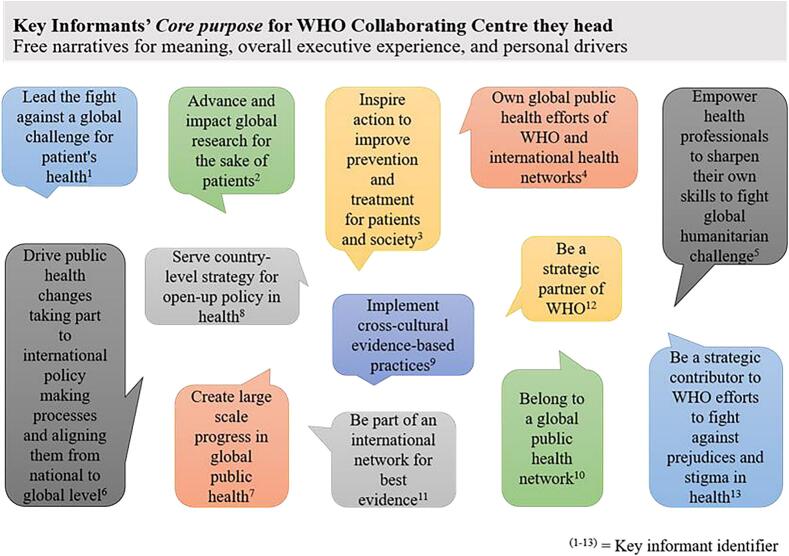
Core Purposes of Directors for their WHOCCs (n=13).

Directors spontaneously reported elements reflecting their **core purpose** for leading a WHO CC (Fig. 1) The figure highlights the unique motivations and values that drive their leadership. Core purposes were particular to each of them and constituted their “why” for leading a WHO CC. They all reported that this meaningful purpose helped them overcome numerous daily challenges in their mission.

**Fig. 2:**
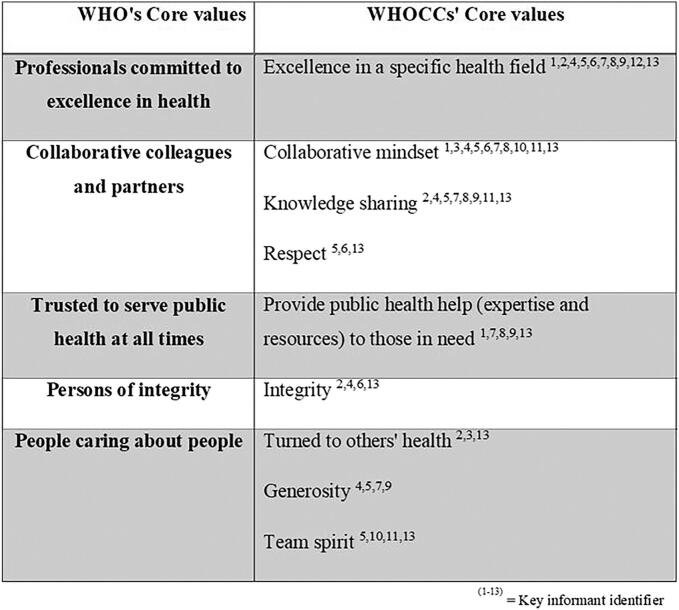
Extent of alignment of the Core Values of CCs Directors with WHO’s Core Values (n=13).

During the interviews, directors were able to freely provide several **core values** in their duty as heads of WHO CCs. (Fig. 2). They all exhibited at least one shared core value with WHO, most aligned with at least three of the five WHO values, and one WHO CC shared them all. The most shared core values were “*professionals committed to excellence in health*” and “*collaborative colleagues and partners*”.

**Fig. 3:**
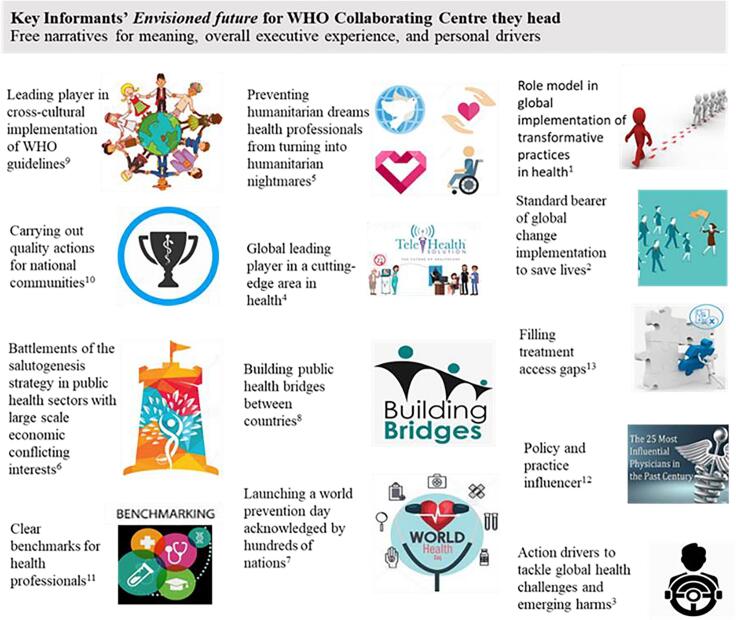
**Envisioned Future of CCs’ Directors (n=13)** *visual icons represent the unique Big Hairy Audacious Goal (BHAG) of each CC.

Open narratives allowed for the identification of each of the 13 directors’ **envisioned futures** for their WHO CCs. They corresponded to BHAGs, with vivid descriptions provided for each. The aims are reflected in both text and visual icons corresponding to the directors’ statements (Fig. 3).

The most accurate strategic positioning for WHO CCs was found to be the one based on benefits to public health and clinical practice. Directors reported that WHO CCs constitute key elements in decision-making within the health sector, particularly in public health and clinical care, at both global and country levels. According to their experience, directors also reported that the commonly misconceived natural attractors for working in WHO CCs (such as the label and career progression) do not hold true in practice. Indeed, WHO CCs’ collaborators were reported to be usually attracted by the international recognition of a research team, by a particular health topic or by a pioneering scientist working in the WHO CC rather than by the WHO CC label itself. Directors advised that if career advancement is to be used as an effective strategic positioning argument, structural efforts are needed at the organizational level.

### 3.3 Recommendations: actionable strategies derived from expert insights

In addition to important conceptual findings for the management and strategic directions of WHO CCs presented above, the present work yielded concrete and practical field-based recommendations (Table 2, Table 3, Table 4, Table 5).

**Table 2:** Reported threats to the enduring existence of WHO CCs (n = 13).

**Category**	**Threats**	**Key Informant Identifier**
**Operational**	Additional workload (no designated time for WHO CC tasks)	1, 6
	Slow progress of working groups' processes	1, 2, 4
	Demands under time pressure	1, 2, 6
	Cumbersome collaborative regulations	1, 2, 4, 5, 6
	Forces dispersion in too large scopes of collaboration	1
**Resource**	Lack of resources (financial and human)	1, 2, 6
	Lack of wherewithal to act (resources, information, regulations)	1, 4, 6, 7, 8, 9, 10, 11, 12, 13
**Collaborative**	WHO organizational model operating in silos and lacking crosscutting needed for some projects	1, 2, 4
	Politically driven context of action	1, 2
	Perceived one-way collaborative framework with WHO rather than partnership framework	2, 4, 6
	Perceived lack of recognition of positive contributions from WHO counterparts	2, 6, 10, 11
	Perceived lack of value given by WHO counterparts to human resources, time, and expertise	2, 5, 6
	Lack of integration in the strategic vision of the host institution	1, 4, 5, 8, 10
**Contextual**	Lack of country-level adherence and acknowledgment	3, 4, 8, 9, 10, 11
	Lack of interest from medical staff and schools	3, 13
	Lack of societal awareness	3, 6, 13
	Challenging nosography update for emerging harms	3
	Lack of succession for continuity of collaborative status and action	3, 5, 6
	Misconception of WHO CC's mainspring	3, 7

**Table 3:** Key actions at different levels (internal, organizational, and external) which should be taken into account to ensure the long-term viability of WHO CCs (n = 12).

**Level of action**	**Key actions needed for enduring existence of WHOCCS**	**Key Informant identifier**
Internal level *WHOCC*	Realize the activities committed to with WHO	3, 6, 7, 9, 13
	Think and act as an ambassador of both the hosting institution and WHO	5, 6, 13
	Motivate people working in health sector to undergo WHO CC activities	2, 6, 13
	Commit to scientific-evidence based information production	5, 9, 11
	Network with international colleagues and other centres	3, 8, 10, 13
	Develop a strategic plan at 4, 8 and 12 years	1
Organizational level *Hosting institution*	Include WHO CC in institution’s strategic vision	1, 2, 4, 10, 13
	Have an organizational governance for the collaboration with WHO: top management of a hospital, deanship of a University	4
	Be tangibly implicated: consider WHO CC as an additional department of the institution and part of institutional communication and reporting	2, 4, 5, 9, 13
	Revise periodically with institutional governance the collaboration status, achievements and issues	4, 5, 13
	Breakdown the strategic goals related to WHO CCs into strategic projects (start, end, indicators of success, governance)	4
	Make resources available and flexible to achieve specific WHO CC projects	5, 6, 7, 8, 10, 11, 13
	Understand and acknowledge the important role of WHO at international stage of the health field	3, 7, 9, 13
External level *WHO*	Negotiate at top governance (institution and WHO) a joint-venture with WHO rather than unilateral support	1, 4, 6, 8
	Revise periodically with WHO strategic orientations for WHO CC, common achievements and needed adjustments	1, 2, 4, 6, 13
	Support from Regional office of WHO at a country-level (ministry of health	6, 8, 9, 10, 13

**Table 4:** Strategic drivers displayed by WHO CC Directors (n = 12).

Strategic drivers of hosting institution	Key Informant identifier
Support to WHO providing renowned expertise	1, 9, 13
Large-scale visibility and impact on the health sector	2, 5, 8
Concrete contribution through working with WHO tackling a health issue, to the country-level awareness and measures	3, 8, 9, 13
High-level research opportunities for universities and medical schools to be involved in	3, 7, 8
Contribution to Sustainable Development Goals in a specific health topic	4, 13
Scientific reputation and credibility	2, 5, 8, 9, 13
Orientation towards community solutions	6, 7, 10
Willingness to help disadvantaged populations	4, 7, 9
International cooperation player	1, 2, 4, 8, 9, 10, 11

**Table 5:** Key leadership skills that were identified as necessary to lead WHO CCs, categorized using the conceptual framework (n = 12).

Practices	Emotional intelligence components
Asking “What needs to be done?”^4,5,6,13^	Self-awareness^2^
Asking “What is right for the organization?”^2,4,5,6,13^	Self-regulation^2,4,5^
Developing and revising action plans^2,5,6,8,13^	Motivation^1,3,6,10^
Taking responsibility for decisions^1,2,3,6,13^	Empathy^2,3,5,9,10^
Taking responsibility for communication^1,3,5,6,8,11,13^	Social skills^2,3,4,5,6,7,8,9,10,13^
Running productive meetings ^4,13^	
Thinking and saying We rather than I^5^	

**The threats** identified by the directors highlight the multifaceted challenges faced by WHO CCs in their collaboration with WHO and their host institutions (Table 2). They could be grouped into four different categories:

1. **Operational Threats**: Focus on workload, process inefficiencies, and time pressures.
2. **Resource Threats**: Highlight lack of financial, human, and informational resources.
3. **Collaborative Threats**: Emphasize issues related to WHO’s organizational structure, political context, and recognition gaps.
4. **Contextual Threats**: Address broader societal, institutional, and systemic challenges.

Addressing these threats requires a comprehensive approach that includes operational, resource, collaborative, contextual, and specific operational strategies. By implementing the suggested **mitigation strategies**, WHO CCs can enhance their sustainability and effectiveness in supporting global health efforts (Table 2).

The **key actions** needed to ensure the lasting existence of WHO CCs, as identified by the directors, are outlined in Table 3. They highlight the multifaceted approach required to ensure the long term viability of WHO CCs. These actions span internal, organizational, and external levels, addressing various aspects of collaboration, resource management, strategic planning, and governance.

**The strategic drivers** that host institutions should prioritize to ensure the continued existence and effectiveness of WHO CCs are outlined in Table 4. These drivers include: support to WHO, visibility, contribution to health issues and research, reputation and credibility, community and population focus, and international cooperation.

**The effective leadership practices** identified by the directors (Table 5) highlight the importance of emotional intelligence in effective leadership within WHO CCs. These practices span self-awareness, self-regulation, motivation, empathy, and social skills. They each contribute to a leader's ability to drive progress, achieve results, and build strong relationships within the organization.

The involvement of all stakeholders, including WHO, host institutions, and WHO CCs themselves, is crucial for the successful implementation of the aforementioned leadership practices, drivers and actions as well as for the achievement of the workplan goals.

For a WHO CC to overcome various management challenges, the following **operational solutions** were co-constructed with expert leaders worldwide and cover two critical issues: financial and communication.

A- How to mitigate a WHO CC’s financial issues.

a- **WHO CC & hosting institution**
- Secure basic financial resources for the functioning of WHO CCs.
- Use some of the WHO CCs’ activities (trainings, expertise tasks, research, and/or funding) to generate financial outcomes to cover some of the expenses.
- Open funding sources at the host organization that WHO CCs could go to for funding certain projects.
- Control for financial conflicts of interest for WHO CCs and for the host institution (funding from industrial entities involved in health related products).
b- **WHO & WHO CCs**
- Engage industrial entities as partners in global projects under the auspices of WHO, whenever possible: ensuring a charter that controls for conflicts of interest and guarantees representativeness, equity, and diversity.
- Support from WHO at the country-level, highlighting the critical role of WHO CCs and the importance of country-level backing, including financially whenever possible.
c- **WHO CCs & external partners**
- Seek financial support from country-level public health authorities by showing them the concrete applications of some WHO CC activities at the country-level.
- Some research funds might be influenced by the WHO CC label and its corresponding activities.

B-How to manage communication issues specific to WHO and WHO CCs.

- Enhance the ability of WHO CCs to act as WHO ambassadors and to be credited with reliable and updated information on global efforts within a particular health area: provide timely information on collaboration topics to WHO CCs whose scope is impacted. Giving WHO CC executives access to certain tagged information available online in WHO system (such as keywords defining their scope) could be a zero cost option. The requirement would thus be to fully respect WHO confidentiality levels of information sharing.
- Fight overlapping public health efforts (global, regional, and cross-cutting working groups): communicate with impacted WHO CCs and all WHO departments and offices about ongoing WHO activities in specific health topics or goals, in order to foster synergies and create coherence. The previously proposed tagged information system could also be interesting.
- Sustain current WHO CCs’ motivation and inspire organizations to collaborate with WHO through increased public acknowledgement. Regular posts on a dedicated webpage highlighting the major achievements accomplished with support from the different WHO CCs could be an effective and low-cost way to increase visibility.

## 4 Discussion

### 4.1 Key findings

a. **Core Purpose and Values:**
  • WHO CCs Directors reported alignment with WHO’s core values (e.g., “commitment to excellence,” “collaboration”).
  • Core purposes varied but centered around global health impact (e.g., “advancing equity in mental health”).
b. **Strategic Positioning:**
  • WHO CCs were perceived as key players in global health decision-making but faced misconceptions (e.g., label attractiveness vs. actual impact).
  • WHO CCs Directors advised structural changes to leverage WHO CCs for career advancement (e.g., integrating WHO CCs’ activities into academic promotions for WHO CCs’ staff).
c. **Threats to Sustainability:**
  a. Lack of resources (financial/human) and recognition from WHO.
  b. Communication gaps and cumbersome regulations.
d. **Leadership Practices:**

Effective WHO CCs Directors exhibited self-awareness, strategic vision, and empathy.

## 5 Strengths of the study

This study is notable for its evidence-based contributions to the field of global public health. The role of WHO CCs is significant in global public health efforts, as they provide scientific expertise to WHO, which translates into public health recommendations for its member states. The present study contributes to the enhancement of the sustainability and impact of WHO CCs.

The study explores the potential of applying management science theoretical frameworks to address the specific challenges encountered by WHO CCs, namely leadership development, resource allocation, effective communication, and their respective frameworks and regulations. This transition from theoretical concepts to practical challenges requires a qualitative research approach that aligns the Conceptual Framework with Empirical Observations (based on data collection from interviews with WHO CCs directors including their reasoned opinions and expressed needs).

The study also acknowledges the important role of the leadership of WHO CCs directors in organizational-level strategic thinking, adaptability, and high standard practices. The present study elucidates eight such practices that are of considerable significance. Finally, the issue of usability is important. A significant strength of the study lies in its practical application, offering actionable recommendations that can effectively and efficiently enhance the sustainability of WHO CCs in addressing global public health challenges.

## 6 Limitations

*Small sample size* (n = 13) limits generalizability, however this is a qualitative not a quantitative design.

*Self-reported data may introduce recall or social desirability bias.* Indeed, the main limitation of qualitative research is represented by subjectivity bias since the researchers share the same professional background. However, this is not necessarily negative as it was a necessary condition to understand the topic at hand. It was essential to understand its complexity and boundaries, and to foster a trustworthy environment for fellow workers to share the richness of their experiences and knowledge. *Limited thematic scope*: This study addresses only a few thematic issues related to leadership and management challenges faced by WHO CCs. Further research is needed to explore additional themes and perspectives.

*Lack of WHO’s perspective as a key stakeholder:* Another limitation is the partially blind navigation of topics related to major stakeholder's perspectives, WHO, and decision-making records, beyond what was available online or already known by the interviewed WHO CC directors.

Lastly, recommendations are *context-specific* and should be adapted to regional/institutional nuances.

## 7 Policy Implications

Our study suggests the need for developing leadership training programs for WHO CC directors; for integrating WHO CC activities into host institutions’ strategic plans with dedicated funding. Meanwhile, WHO CC’s directors should adopt ambassadorial roles to bridge WHO and local health systems.

## 8 Conclusion

The sustainability of WHO CCs requires a comprehensive approach that integrates strategic management, effective leadership, and operational solutions to address financial and communication challenges. By implementing the recommended actions and involving all stakeholders, including WHO, host institutions, and WHO CCs themselves, we can enhance the sustainability, effectiveness, and impact of WHO CCs in supporting global health efforts. This collaborative approach is essential for addressing the complex challenges faced by WHO CCs and ensuring their continued contribution to global public health.

WHO CCs are indispensable to global health but face sustainability challenges. This study offers (a) **Diverse perspectives** (five WHO regions, multiple institution types); (b) **Practical recommendations** grounded on empirical data; and (c) **A novel focus on leadership** as a driver for WHO CC sustainability.

Study findings highlight the need for: (i) **Structured strategic management** (alignment with WHO’s goals, resource allocation); (ii) **Leadership development** (self-awareness, strategic positioning); and (iii) **Improved communication** (WHO- WHO CCs synergies, public acknowledgment). By implementing these recommendations, stakeholders can enhance WHO CCs’ sustainability and impact on global health.

## 9 Research in context

1. What is already known about the topic?

WHO Collaborating Centres (WHO CCs) are recognized for their crucial role in global public health, providing scientific and technical support in specific health sectors. They contribute to WHO program implementation, guideline development, disease monitoring, and training. However, sustaining them is challenging due to their dual affiliation (WHO and host institutions). The decreasing number of WHO CCs highlights significant losses in crucial expertise for large-scale collaboration.

2. What does this study add to the literature?

This study identifies strategic, and management challenges facing WHO CCs and emphasizes the importance of leadership skills for their sustainability. Through interviews with experienced WHO CC directors, the study provides empirical insights into effective leadership practices, core values, and envisioned futures aligned with WHO's goals. It offers practical recommendations to address financial and communication challenges, enhancing WHO CCs’ sustainability and impact.

3. What are the policy implications?

The findings inform policies that can focus on leadership development, resource allocation, and effective communication. Enhancing the ability of WHO CCs to act as WHO ambassadors and ensuring timely information sharing, can strengthen global health collaboration. These insights support the development of targeted interventions to sustain and amplify the impact of WHO CCs in global public health efforts.

## CRediT authorship contribution statement

**Sophia Achab:** Writing – review & editing, Writing – original draft, Project administration, Method, ology, Investigation, Conceptualization. **Benedetto Saraceno:** Writing – review & editing, Supervision, Conceptualization.

## Declaration of competing interest

The authors declare that they have no known competing financial interests or personal relationships that could have appeared to influence the work reported in this paper.
