# Supplementary material for: Strengthening global health cooperation-insights from worldwide WHO collaborating centres
Source: Health Policy Open. 2025 Dec 19;10:100158. doi: 10.1016/j.hpopen.2025.100158 (PMC12811637; doi:10.1016/j.hpopen.2025.100158)
Supplement: Supplementary Data 1 [file mmc1.docx]

**Appendix**

**01- Interview guide for in-person and phone participants**

*Introduction:* I would like to discuss your experience of Management of a WHO Collaborating Center. I’m here to learn from your experience as you are an expert here. The interview will take about 1 hour. Do you mind if I record our conversation because it’s very important not to miss anything? There are no correct or wrong answers; your views and experience are what matter.

***General background information****:*

I’d like to start by asking you a few general background questions:

*Q1.* What is your past and current management position at your WHO Collaborating center?

*Q2.* For how many years the center you are managing has been designated as WHO Collaborating center?

***Free narrative***

*Q3.* What does the term WHO Collaborating center means for you?

*Q4.* Can you please describe your overall experience in the management of your WHO Collaborating center?

*Q5.* What do you consider as important drivers for you in your management position at your WHO Collaborating center?

***Key questions***

Now I would like to know more about your impression and experience with regards to a WHO Collaborating center management.

In your opinion,

*Q6.* What should be the strategic drivers for a health institution to develop a WHO Collaborating center?

*Q7.* Which would be the key actions from a management perspective that should be taken (by the Director and /or by the hosting health institution) to support WHO Collaborating center evergreen existence?

**Round 2**

Based on your experience,

*Q8.* Does the label WHO Collaborating center attract collaborators to its hosting health institution?

*Q9.* Does participating to WHO collaborating center activities, contribute to the academic career progression of staff members?

- If yes, How?

- If no, Should it be the case?

*Q10.* Does WHO collaborating center activities, contribute to decision making in the health field?

If yes, for which level/levels (please provide 2-3 concrete examples for each level):

- National?

- Global?

- Clinical?

- Public health?

If no, should it be the case? Why?

*Q11*. What are the key leadership skills a WHO collaborating center manager should have?

*Q12.* Are project management skills important for WHO collaborating center management? Why?

*Q13.* Are financial considerations important for the management of a WHO collaborating center? Why?

*Q14*. Is Communication a key issue for the management of a WHO collaborating center? Why?

Conclusion

*Q15.* Based on you experience and background, what would be the 3-5 main recommendations that you would make to a health institution aiming at developing and making its WHO collaborating center sustainable?

Is there anything else you would like to say?

Thank you for your time.

**02- Self-administered questionnaire for email participants**

**Round 1**

***Introduction:*** I would like to discuss your experience of Management of a WHO Collaborating Center. I’m here to learn from your experience as you are an expert here. The interview will take about 1 hour. Do you mind if I record our conversation because it’s very important not to miss anything? There are no correct or wrong answers; your views and experience are what matter.

***General background information***

I’d like to start by asking you a few general background questions:

*Q1.* What is your past and current management position at your WHO Collaborating center?

*Q2.* For how many years the center you are managing has been designated as WHO Collaborating center?

***Free narrative***

*Q3.* What does the term WHO Collaborating center means for you?

*Q4.* Can you please describe your overall experience in the management of your WHO Collaborating center?

*Q5.* What do you consider as important drivers for you in your management position at your WHO Collaborating center?

***Key questions***

Now I would like to know more about your impression and experience with regards to a WHO Collaborating center management.

In your opinion,

*Q6.* What should be the strategic drivers for a health institution to develop a WHO Collaborating center?

*Q7.* Which would be the key actions from a management perspective that should be taken (by the Director and /or by the hosting health institution) to support WHO Collaborating center evergreen existence?

**Round 2**

Based on your experience,

*Q8.* Does the label WHO Collaborating center attract collaborators to its hosting health institution?

*Q9.* Does participating to WHO collaborating center activities, contribute to the academic career progression of staff members?

- If yes, How?

- If no, Should it be the case?

*Q10.* Does WHO collaborating center activities, contribute to decision making in the health field?

If yes, for which level/levels (please provide 2-3 concrete examples for each level):

- National?

- Global?

- Clinical?

- Public health?

If no, should it be the case? Why?

*Q11*. What are the key leadership skills a WHO collaborating center manager should have?

*Q12.* Are project management skills important for WHO collaborating center management? Why?

*Q13.* Are financial considerations important for the management of a WHO collaborating center? Why?

*Q14*. Is Communication a key issue for the management of a WHO collaborating center? Why?

Conclusion

*Q15.* Based on you experience and background, what would be the 3-5 main recommendations that you would make to a health institution aiming at developing and making its WHO collaborating center sustainable?

Is there anything else you would like to say?

Thank you for your time.
