# Supplementary material for: Strengthening global health cooperation-insights from worldwide WHO collaborating centres
Source: Health Policy Open. 2025 Dec 19;10:100158. doi: 10.1016/j.hpopen.2025.100158 (PMC12811637; doi:10.1016/j.hpopen.2025.100158)

Figure 1: Core Purposes of Directors for their WHOCCs (n=13)

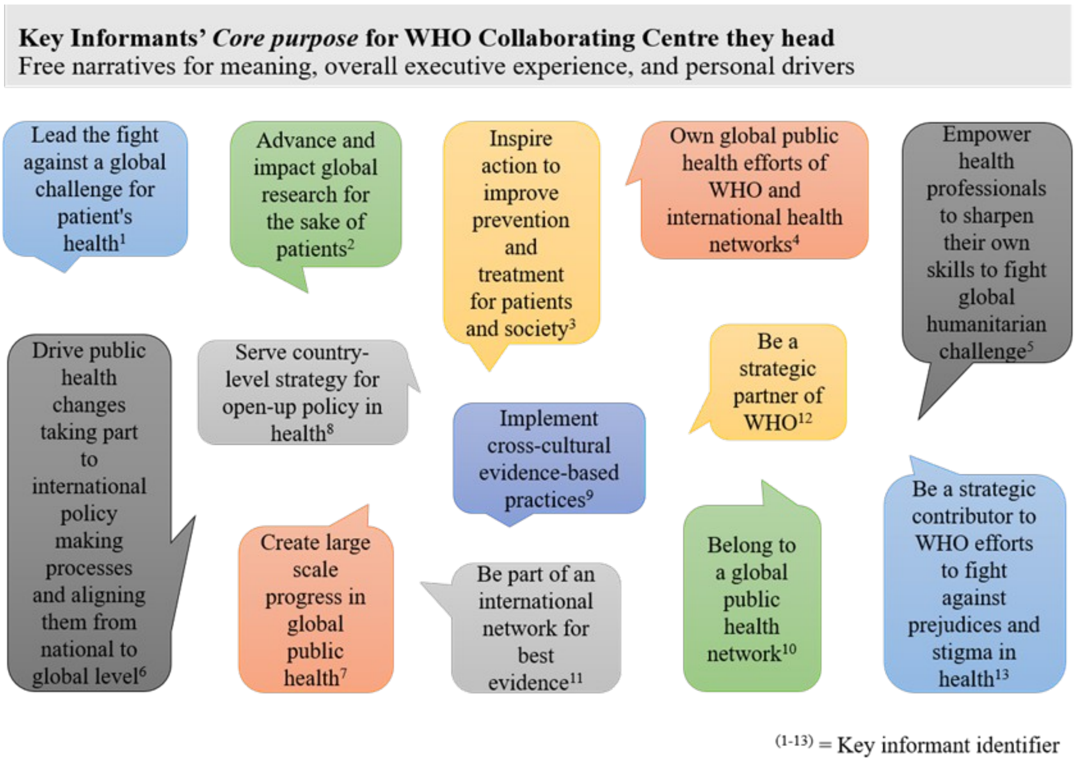

Figure 2: Extent of alignment of the Core Values of CCs Directors with WHO's Core Values (n=13)

WHO's Core values	WHOCCs' Core values
**Professionals committed to excellence in health**	Excellence in a specific health field [1,2,4,5,6,7,8,9,12,13]
**Collaborative colleagues and partners**	Collaborative mindset [1,3,4,5,6,7,8,10,11,13]<br>Knowledge sharing [2,4,5,7,8,9,11,13]<br>Respect [5,6,13]
**Trusted to serve public health at all times**	Provide public health help (expertise and resources) to those in need [1,7,8,9,13]
**Persons of integrity**	Integrity [2,4,6,13]
**People caring about people**	Turned to others' health [2,3,13]<br>Generosity [4,5,7,9]<br>Team spirit [5,10,11,13]

[(1-13)] = Key informant identifier

Figure 3: Envisioned Future of CCs' Directors (n=13) **visual icons represent the unique Big Hairy Audacious Goal (BHAG) of each CC*

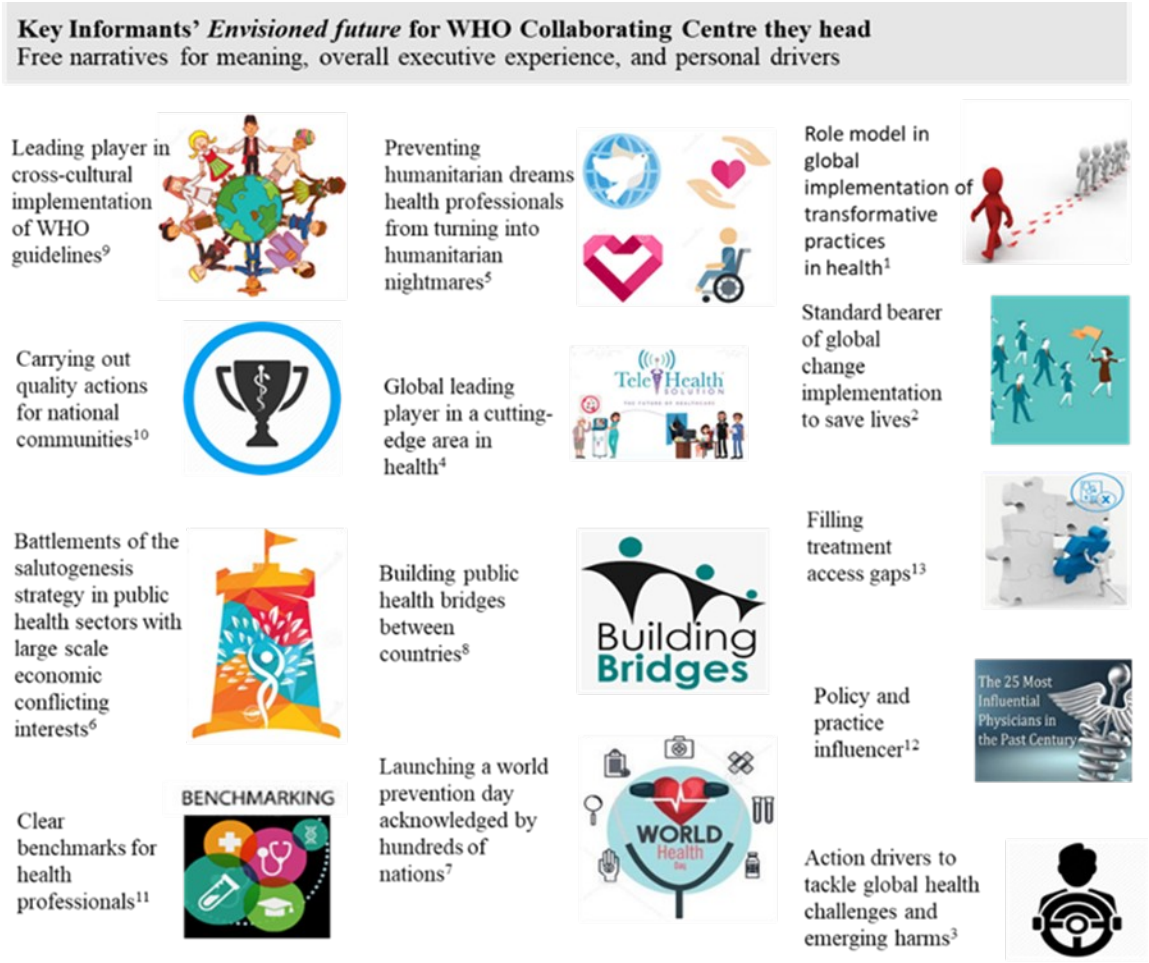

Supplement: Supplementary file 2 [file mmc2.docx]
